# GnRH level regulation in the hypothalamus of female rats of different age

**DOI:** 10.1186/2193-1801-4-S1-P18

**Published:** 2015-06-12

**Authors:** Andrew Korenevsky, Alexander Arutjunyan, Yulia Milyutina, Gleb Kerkeshko, Michael Stepanov, Irina Zaloznyaya

**Affiliations:** D.O. Ott Institute of Obstetrics, Gynecology and Reproductology, Russian Federation

**Keywords:** GnRH, biogenic amines, hypothalamus

In recent years the study of age-related derangements of the gonadotropin releasing hormone (GnRH) synthesis and secretion resulting from both the GnRH gene expression changes and interaction of glia with GnRH-ergic neurons of the hypothalamus is a focus of attention. It was demonstrated earlier that this could result from the decreased activity of monoaminergic and peptidergic systems that control the GnRH preovulatory secretion surge initiation, specifically from the loss of the signal coming from the suprachiasmatic nuclei (SCN) of the hypothalamus. This signal is critical for the emerging of the GnRH regular cyclic secretion, which is mediated prominently by vasoactive intestinal peptide (VIP). We have studied age-related changes in the biogenic amines and VIP content in the hypothalamic structures responsible for the GnRH synthesis and secretion. It has been shown by us that the GnRH level in the median eminence with the arcuate nuclei (ME-Arc) of the hypothalamus of 22-month-old rats is half as high compared to that of 7-8-month-old animals. Beside that, the VIP level in the SCN tended to decrease, with the norepinephrine, dopamine, and 5-hydroxyindoleacetic acid levels decreased significantly in the median preoptic area of the hypothalamus responsible for the GnRH synthesis and in the ME-Arc exercising its secretion into the portal vein of the pituitary. It has been shown that the initial phase of the reproductive failure with 13-14-month-old animals having irregular estrous cycles is characterized by gradual disappearance of the normal biogenic amine diurnal dynamics in the studied hypothalamic structures, which could be due to the loss of regulatory signals coming from the SCN.

